# Draft Genome Sequence of *Mycobacterium bovis* Strain AN5, Used for Production of Purified Protein Derivative

**DOI:** 10.1128/genomeA.00277-14

**Published:** 2014-04-03

**Authors:** Ana Beatriz Canevari Castelão, Christiane Nishibe, André Moura, Andrea Padilha de Alencar, Mariana de Azevedo Issa, Mikael Arrais Hodon, Pedro Moacyr Pinto Coelho Mota, Érica Bravo Sales, Antônio Augusto Fonseca Júnior, Nalvo Franco Almeida, Flábio Ribeiro Araújo

**Affiliations:** a Programa de Pós-Graduação em Ciência Animal, UFMS, Campo Grande, Mato Grosso do Sul, Brazil; b School of Computing, UFMS, Campo Grande, Mato Grosso do Sul, Brazil; c Laboratório Nacional Agropecuário–LANAGRO/MG, Ministério da Agricultura, Pecuária e Abastecimento, Pedro Leopoldo, Minas Gerais, Brazil; d Embrapa Beef Cattle, Campo Grande, Mato Grosso do Sul, Brazil

## Abstract

*Mycobacterium bovis* strain AN5 has been used to produce purified protein derivative (PPD) for the intradermal test for bovine tuberculosis since it was introduced in 1948. This work reports the draft genome sequence of *M. bovis* AN5, which is used for the production of bovine PPD in Brazil, as well as comparisons to other strains of *M. bovis* and *Mycobacterium tuberculosis*.

## GENOME ANNOUNCEMENT

*Mycobacterium bovis* is the causative agent of bovine tuberculosis, a disease that accounts for annual losses of $3 billion (1) and that poses a threat to public health and animal welfare (2, 3). In Brazil and many other countries, the program for controlling bovine tuberculosis involves testing cattle with an antigen preparation of the *M. bovis* AN5 strain, namely, purified protein derivative (PPD), followed by slaughtering positive reactors. This strain produces a high yield of cell mass on glycerinated medium, a desirable phenotype that was selected by repeated subculture of the bacillus on laboratory medium (4). The draft genome of the *M. bovis* AN5 strain used in Brazil for PPD production is reported in this paper. Comparisons of the genes coding for PPD proteins in other strains of *M. bovis* (AF2122/97 and 04-303) and *Mycobacterium tuberculosis* H37Rv are also described.

The genome sequence was obtained using MiSeq technology (5), producing a total of 3,240,633 paired-end reads. After filtering, 2,269,762 paired-end reads and 372,399 single-end reads were used in the assembly. We performed a reference-assisted genome assembly of the filtered data for *M. bovis* AN5 with the *M. bovis* AF2122/97 strain (GenBank accession no. NC_002945) using Bowtie (6). The assembly contains 70 contigs (with sizes no shorter than 500 bp) and an *N*_50_ of 150,219. Annotation for the *M. bovis* AN5 genome was obtained by using the NCBI Prokaryotic Genome Annotation Pipeline (7) and comprises 3,938 coding sequences (CDSs) and 48 RNA genes (3 rRNAs and 45 tRNAs). Out of the 3,938 CDSs, 2,830 (72%) have a functional assignment.

A comparison of the genes coding for proteins found in bovine PPDs produced in the United Kingdom (PPDUK) and in Brazil (PPDBR) by liquid chromatography-tandem mass spectrometry revealed a total of 116 proteins. Of these, 67 were found only in PPDUK, 12 were found only in PPDBR, and 37 were identified in both samples (8). This fact suggests that the Brazilian *M. bovis* AN5 isolate might be missing some PPD genes or that the genes contain a high degree of variation. Genes coding for all 116 proteins found in both PPDs were compared to their orthologs in *M. bovis* AF2122/97 (1), *M. bovis* 04-303 (9), and *M. tuberculosis* H37Rv (10) and showed a high degree of conservation, with identities ranging from 98% to 100%. In addition, no frameshifts were detected. Genes coding for the immunodominant proteins MPB64, MPB70, MPB83, GroEL, GroES, HspX, CFP-10, and CFP-21 are 100% conserved among the orthologs. The gene coding for ESAT-6 showed 100% identity with orthologs in *M. tuberculosis* H37Rv and *M. bovis* AF2122/97 strains, and 99% identity with the ortholog in *M. bovis* 04-303. The gene coding for MPB53 showed 100% identity with orthologs in *M. bovis* 04-303 and *M. bovis* AF2122/97 and 99% identity with that in *M. tuberculosis* H37Rv. In conclusion, neither gene deletion nor gene variation in *M. bovis* AN5 strains used for PPDBR supports the proteomic differences compared with PPDUK.

### Nucleotide sequence accession numbers.

This whole-genome shotgun project has been deposited at DDBJ/EMBL/GenBank under accession no. AWPL00000000. The version described in this paper is version AWPL01000000.
